# The *Caenorhabditis elegans* Shugoshin regulates TAC-1 in cilia

**DOI:** 10.1038/s41598-023-36430-8

**Published:** 2023-06-09

**Authors:** R. Reed, K. Park, B. Waddell, T. A. Timbers, C. Li, K. Baxi, R. M. Giacomin, M. R. Leroux, C. E. Carvalho

**Affiliations:** 1 Department of Biology, University of Saskatchewan, Saskatoon, Canada; 2 Department of Molecular Biology & Biochemistry, Simon Fraser University, Burnaby, BC V5A 1S6 Canada; 3 Centre for Cell Biology, Development, and Disease, Simon Fraser University, 8888 University Drive, Burnaby, BC V5A 1S6 Canada; 4 Present Address: Terry Fox Laboratory, BC Cancer, Vancouver, BC V5Z 1L3 Canada; 5 Present Address: Department of Medical Genetics, University of British Columbia, Vancouver, BC V6T 1Z3 Canada

**Keywords:** Genetics, Genetic interaction, Molecular biology

## Abstract

The conserved Shugoshin (SGO) protein family is essential for mediating proper chromosome segregation from yeast to humans but has also been implicated in diverse roles outside of the nucleus. SGO’s roles include inhibiting incorrect spindle attachment in the kinetochore, regulating the spindle assembly checkpoint (SAC), and ensuring centriole cohesion in the centrosome, all functions that involve different microtubule scaffolding structures in the cell. In *Caenorhabditis elegans*, a species with holocentric chromosomes, SGO-1 is not required for cohesin protection or spindle attachment but appears important for licensing meiotic recombination. Here we provide the first functional evidence that in *C. elegans*, Shugoshin functions in another extranuclear, microtubule-based structure, the primary cilium. We identify the centrosomal and microtubule-regulating transforming acidic coiled-coil protein, TACC/TAC-1, which also localizes to the basal body, as an SGO-1 binding protein. Genetic analyses indicate that TAC-1 activity must be maintained below a threshold at the ciliary base for correct cilia function, and that SGO-1 likely participates in constraining TAC-1 to the basal body by influencing the function of the transition zone ‘ciliary gate’. This research expands our understanding of cellular functions of Shugoshin proteins and contributes to the growing examples of overlap between kinetochore, centrosome and cilia proteomes.

## Introduction

The conserved protein family, Shugoshin (SGO), plays an integral role in cellular division, and has been more recently implicated in extranuclear functions. In species with a defined centromere (termed monocentric), during cell division SGO participates in centralizing chromosome attachment to the spindle as well as cohesin protection, both processes which require a functional member of the protein family. Prior to anaphase entry in mitosis and meiosis, sister chromatid association (and thus genomic integrity) is maintained by the cohesin complex, which is in turn guarded by SGO. Shugoshin accomplishes this by recruiting Protein Phosphatase 2A (PP2A) to the centromere to dephosphorylate cohesin’s kleisin subunit^1,2^. Upon bipolar attachment of chromosomes to the spindle and the generation of sufficient tension, Shugoshin is displaced from the centromere, rendering centromeric cohesin vulnerable to phosphorylation and subsequent cleavage by separase, ultimately allowing sister chromatids to part^3^. Besides Shugoshin proteins’ role in cohesin protection, they also participate in spindle attachment by recruiting the chromosome passenger complex (CPC)^4^. The CPC is responsible for executing error correction of kinetochore-microtubule attachments and removes microtubules that have failed to generate tension^5^. If this occurs, the Spindle Assembly Checkpoint (SAC) is activated and cell-cycle progression is delayed^6^. SGO links the CPC and SAC by interacting with the Mad2 subunit of the SAC and localizing the two complexes together at the centromere^1^. Thus, Shugoshin serves as an adaptor protein in protecting sister chromatid cohesion in mitosis and meiosis and as an essential regulator of the SAC.

Despite SGO’s role in chromosomal segregation, these versatile proteins are also implicated in non-canonical roles outside the nucleus. In humans, a shorter splice variant of human *Sgo1*, *sSgo1*, localizes to the centrosome and is proposed to protect centriolar cohesion during mitosis^7^. Extranuclear functions of vertebrate SGO proteins are further supported by the recent discovery of its role in regulating pacemaker activity in the cell membranes of myocytes and its involvement in Chronic Atrial and Intestinal Dysrhythmia Syndrome (CAID)^8^. Consistent with post-mitotic functions for this protein family, mouse SGO1 is expressed in distinct terminally differentiated cells in neonatal mice, including neurons^9,10^.

In contrast to monocentric organisms with a defined centromere, holocentric organisms such as *Caenorhabditis elegans* possess a diffuse kinetochore with microtubule attachments occurring along the chromosome length^11^. Although the sole *C.* *elegans* Shugoshin homolog, SGO-1, is present at the spindle and dividing chromosomes, it is dispensable for cohesin protection and SAC function unlike in monocentric organisms^12–14^. Instead, *C. elegans* appears to utilize SGO-1-independent control mechanisms to survey microtubule attachment at the kinetochore and prevent premature sister chromatid disjunction. This raises the intriguing possibility that the worm protein may be involved in previously undescribed but evolutionarily conserved functions^15–17^. Consistent with this idea, an earlier meiotic role of SGO-1 has been described in prophase I in *C. elegans*, where SGO-1 is required for checkpoint activity and contributes to ensuring meiotic axis function^18^.

Although Shugoshin is implicated in a variety of functions, they are all underscored by microtubule interactions. Here we present evidence that SGO-1 is expressed in terminally differentiated sensory neurons in *C. elegans*. SGO-1 concentrates at the base of cilia, yet another microtubule-based structure. Moreover, we show that SGO-1 is a binding partner of the known microtubule regulator, TAC-1. In *C. elegans*, cilia play a critical role in sensory organ functions that coordinate behavioural and developmental processes and are present as a single projection from the surface of most vertebrate cell types^19^. The cilium originates from the centriolar basal body (BB), where microtubules polymerize into the axoneme^20^. Distal from the BB lies the transition zone (TZ), a crucial region for cilia compartmentalization that regulates the passage of proteins in and out of this organelle. Deficiencies in ciliary formation, structure and/or function are behind a pleiotropic group of disorders termed ciliopathies, many of which map to mutations in proteins that localize to the BB, TZ, or an intraflagellar transport (IFT) system that shuttles proteins into and out of cilia^21^. Our study suggests that an appropriate threshold of SGO-1/TAC-1 activity is required for the proper functioning of cilia in *C. elegans*, laying the groundwork for a more mechanistic understanding of their functional interaction.

## Results

### SGO-1 localizes to the basal body-ciliary organelle

**Figure 1:**
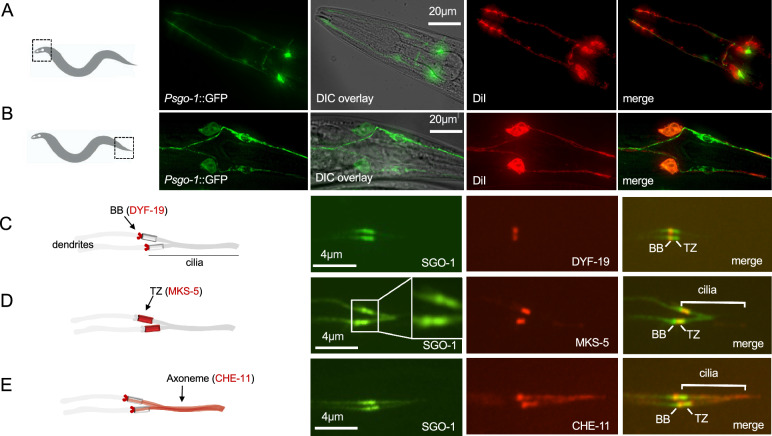
The *C. elegans* Shugoshin is expressed in sensory neurons and localizes to cilia. Expression of *Psgo-1::GFP* in ciliated neurons of the head (**A**) and tail (**B**) stained by DiI. Microscopy images of *C. elegans* are oriented such that the left is anterior and the right posterior. (**C**, **D**, **E**). Localization of SGO-1 in different ciliary domains of the phasmid neurons (indicated in the diagrams on the left). (**C**) SGO-1::GFP localizes to the basal body (BB) with DYF-19. (**D**) SGO-1 co-localizes with MKS-5 in the transition zone (TZ). (**E**) SGO-1::GFP accumulates at the base of cilia and is detectable along the axoneme together with the IFT protein CHE-11.

We set out to investigate alternative functions of SGO-1 in *C. elegans* by determining whether this gene is expressed in terminally differentiated cells in the adult worm. The *sgo-1* gene is the first in a three-gene operon on chromosome IV, which also contains the ecto-apyrase *ntp-1* and an uncharacterized gene, C33H5.13. We created a transcriptional reporter (referred to in this manuscript as *Psgo-1*) driven by 900 bp of sequences upstream of *sgo-1* and carrying the 12 initial codons of exon 1 fused to GFP; this reporter revealed expression in head- and tail-localized sensory neurons (Figs. 1A,B). This is consistent with previous transcriptomic analyses that found an enrichment of *sgo-1* transcripts in *C. elegans* neurons, including in AFD thermosensory neurons, which are part of the amphid sensory organ in the head^22,23^. Closer examination showed that the *Psgo-1::GFP* reporter is expressed in several head and tail neurons, including all 6 pairs of amphid and 2 pairs of phasmid chemosensory neurons that are exposed to the environment and can be filled with the DiI fluorescent dye (Supplementary Figure 1). Since sensory neurons are the only ciliated cells in *C. elegans*, we sought to investigate whether SGO-1 is recruited to the cilium. To this end, we determined the subcellular localization of SGO-1 using a translational SGO-1::GFP reporter expressed exclusively in ciliated neurons via the *osm-5* promoter (*Posm-5*) alongside markers for different cilia domains. We found that SGO-1::GFP accumulates at the dendritic end of sensory neurons, where it co-localizes with the basal body (BB) and transition zone (TZ) markers DYF-19/FBF1 and MKS-5/RPGRPI1L, respectively (Fig. 1C,D). We also noted weaker SGO-1::GFP signal co-localizing with the IFT protein, CHE-11, which is present along the length of ciliary axonemes (Fig. 1E). From these findings, we conclude that the *sgo-1* is expressed in the sensory organs of *C. elegans* and that its protein product, SGO-1, predominantly accumulates within the basal body-ciliary organelle.

### *sgo-1(tm2443)* and *sgo-1(sas02)* mutants have sensory defects with allele-specific differences

**Figure 2:**
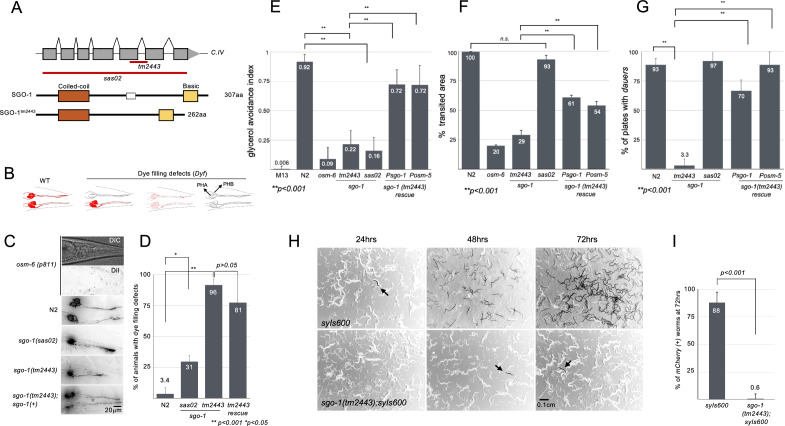
*sgo-1* mutants display developmental and behavioral phenotypes consistent with defects in cilia. (**A**) Diagram of the *sgo-1* (C33H5.15) locus in *C. elegans* and predicted proteins. Deletions used in this work are indicated in red. *sas02* is a gene-edited deletion of the entire genomic region (ATG to stop). *tm2443* is an in-frame 195 bp deletion spanning most of exon 5, all of intron 5 and 15 bp of exon 6, eliminating residues 161 to 208 that is predicted to result in a shorter SGO-1 protein carrying both the coiled-coil and basic domains. The two conserved N (coiled-coil) and C termini (basic) domains are shown. The protein region deleted in *tm2443* is indicated as a white box in the SGO-1 protein. *sgo-1* mutants show cilia-related phenotypes: (**B**) Diagram of dye-filling phenotypes of phasmid neurons in the tail. Red represents Dil uptake. The anterior region is depicted on the left where the two pairs of PHA/B cell bodies are located. Cilia occupy the distal end of dendrites on the right, from where Dil is passively absorbed. (**C**) Representative black-white / inverted DiI-stained images of the tails in N2 and *sgo-1* mutants. (**D**) Quantification of dye filling defects in N2 and *sgo-1* mutants. (**E**) Glycerol avoidance defect. Data represents a n = 30 worms for each genotype, with each worm being tested ten times, averages are plotted. (**F**) Quantification of the roaming defect. Data represents four independent experiments with n = 10 for each genotype. (**G**) Dauer formation defect in *tm2443*. Data represents three independent experiments composed of 10 NGM plates in each. (**H**) Time course of dauer formation on starved plates. Representative black white/inverted images showing sections of 60 mm NGM plates with mix populations of worms carrying the integrated *Pcol-183::mCherry* (*syIs600*) transgene at 24, 48 and 72 h post-starvation at 20 °C. Arrows indicate worms with mCherry expression (dark). (**I**) Quantification of dauer formation at 72 h post-starvation. Plates were washed and resuspended in 200 μl of M9. 15 μl of the worm solution was placed on glass slides and after addition of 10 μl of 20 mM sodium azide, worms were scored for *Pcol-183::mCherry* expression under a UV stereoscope. Each experiment consisted of 5 independent plates (n = 5) and the experiment was repeated 2 times for a total n = 1080 worms per genotype.

We pondered that the ciliary localization of SGO-1::GFP predicted a novel sensory function for Shugoshin, prompting us to check whether depletion of SGO-1 is associated with cilia-related sensory defects. To test this, we used a previously described deletion allele (*tm2443*) as well as a CRISPR/Cas9-generated whole gene deletion and predicted null (*sas02*) (Fig. 2A; Supplementary Figure 2). Shugoshin is largely dispensable for cell division in *C. elegans* and *sgo-1* homozygous mutants are viable and fertile^12^. We first tested the ability of amphid and phasmid neurons in *sgo-1(sas02)* and *sgo-1(tm2443)* animals to passively uptake the lipophilic dye DiI from the environment. A dye-filling defective (Dyf) phenotype is associated with compromised cilia, and thus can be used as an indirect measure of cilia integrity^24^. Compared to N2 worms, *sgo-1(tm2443)* and *sgo-1(sas02)* mutant worms have significant dye filling defects (Dyf phenotype; Fig. 2B-D), suggesting structural compromise in ciliary compartments. We concluded that *sgo-1* mutants are prone to ciliary defects. Such ciliary anomalies can impair the animal’s chemosensory abilities^25^. Accordingly, *tm2443* and *sas02* animals have defects in avoidance behaviours against repellants such as glycerol, SDS, and chloroquine (Fig. 2E; Fig. 4E). We found these defects could be partially overcome by expressing *sgo-1* rescue transgenes, expressed via its native promoter or exclusively in ciliated neurons using the pan-ciliary neuron *osm-5* promoter, *Posm-5* (Fig. 2E). Disruption of cilia-dependent sensory functions can also impact the normal roaming behaviour of *C. elegans* on food plates; a defect referred to as dwelling^26^. Indeed, *sgo-1(tm2443)* worms are dwellers and show restricted foraging behaviour on a standardized food area, a phenotype also rescued by wild-type *sgo-1* transgenes (Fig. 2F). Cilia-mediated signalling is equally essential to sense hormonal cues needed to trigger the dauer developmental program in response to environmental stressors^27^. In starvation assays, we determined that *sgo-1(tm2443)* worms are dauer-defective, and compared to N2, few mutant worms express the early dauer reporter *Pcol-183::mCherry (syIs-600)* at two days post-starvation (Fig. 2H,I). Similar to the chemotaxis rescue experiments, expressing *sgo-1* using its own promoter or the *Posm-5* promoter can rescue this defect (Fig. 2G).

Altogether, our localization and phenotypic analyses situate the *C. elegans* SGO-1 within cilia and supports a role in regulating the sensory functions of cilia. Interestingly, and unlike the *sgo-1(tm2443)* strain, null *sgo-1(sas02)* mutants showed only a mild dye-filling defect and no significant impairment in roaming behaviour or dauer formation (Fig. 2D,E,F,G), suggesting that these phenotypes may be allele-specific.

The difference between the *sgo-1(tm2443)* and *sgo-1(sas02)* alleles was particularly surprising given they both predict a loss of SGO-1 function. To address these differences, we re-sequenced the *tm2443 sgo-1* genomic locus and cDNA in *tm2443* worms. In contrast with the current annotation^13,18^, we found that the *tm2443* allele is an in-frame 195 bp deletion that removes 45 residues (161–208) from the central region of SGO-1 (Supplementary Figure 4). Contrary to previous interpretation, this deletion does not affect the conserved SGO motif in the C-terminus of the protein, which has been implicated in binding to phosphorylated H2A in yeast^28^.

We next considered whether the SGO-1^*tm2443*^ protein could still correctly localize to cilia. Worms expressing a *sgo-1*^*tm2443*^*::mCherry* reporter under the *Posm-5* promoter showed accumulation of SGO-1^*tm2443*^ at the base of cilia (Supplementary Figure 5A) and co-localization with the wild type SGO-1 protein (Supplementary Figure 6), indicating that SGO-1^*tm2443*^ is indeed recruited to cilia. Human Shugoshin has been shown to homodimerize via the conserved N-terminal coiled coil domain to bind PP2A^29^. If the *C. elegans* Shugoshin acted as a dimer in neurons, a dominant-negative SGO-1^*tm2443*^ protein could presumably disrupt SGO-1 function and potentially account for the additional phenotypes in *sgo-1(tm2443)* worms. In this scenario, we would expect *sgo-1(tm2443)/* + heterozygotes to be severely affected for the sensory phenotypes observed in *tm2443/tm2443* worms. However, we find that the roaming behaviour of *sgo-1(tm2443)*/ + heterozygotes is mostly unaffected (Supplementary Figure 5B), suggesting that sufficient SGO-1 activity must remain in these animals to support cilia function.

The differences in intensity of dwelling defects in *tm2443/* + heterozygotes and *tm2443* homozyotes suggested a dose-dependent effect of SGO-1^*tm2443*^ in cilia. To address this, we compared the roaming behaviour of *tm2443/sas02* trans-heterozygotes with that of *tm2443/* + animals as well as homozygous worms for *tm2443* and *sas02*. We found that the roaming defect in *tm2443/sas02* worms is intermediate between *tm2443/* + and *tm2443/tm2443* worms, while the null *sas02* mutants track normally on food plates (Supplementary Figure 5B). These results suggest a quantitative effect of SGO-1^*tm2443*^ loads in disrupting cilia function. If SGO-1^*tm2443*^ can indeed disrupt cilia function in a dose-dependent way, overexpressing this abnormal protein in ciliated neurons of N2 (*sgo-1* + */sgo-1* +) worms should phenocopy the defects observed exclusively in *tm2443* mutants. Accordingly, we found that N2 worms carrying *Posm-5::sgo-1*^*tm2443*^*::mCherry* transgenes show a significant increase in dwelling behaviour compared to siblings that lost the transgenic array (Supplementary Figure 5C), suggesting that a threshold of SGO-1^*tm2443*^/SGO-1^WT^ function ultimately triggers the roaming defect. These results are consistent with the relatively modest rescue of *sgo-1(tm2443)-*specific defects by N2 *sgo-1(* +*)* transgenes (Fig. 2E,F; Supplementary Figure 5B,C) and support the interpretation that *tm2443* is likely a dose-dependent neomorph allele of *sgo-1*. Overall, our phenotypic analyses of *sgo-1* mutants point to a distinct role of SGO-1 in the biogenesis or maintenance of ciliary structures.

### TAC-1 binds SGO-1 and co-localizes with it at the base of cilia

**Figure 3:**
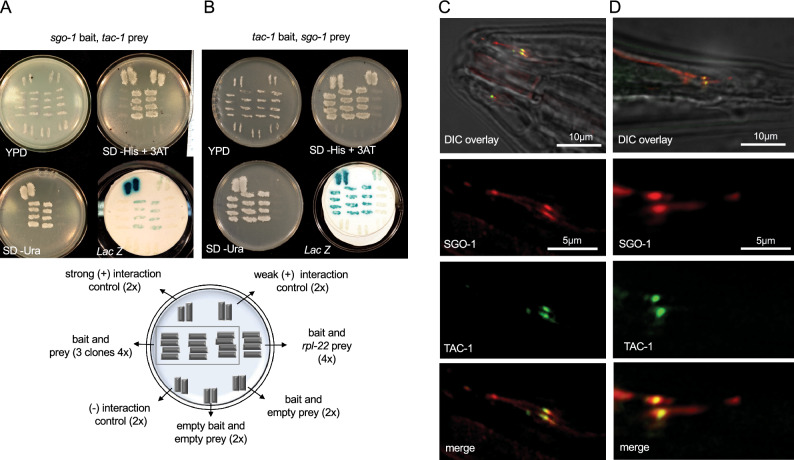
TAC-1 is a SGO-1 interacting protein. (**A**, **B**) SGO-1 and TAC-1 interaction in Yeast Two-Hybrids. Interactions were tested in triplicate using independently transformed yeast clones. Selection of positive interactions was assessed using three assays: Growth in –His + 3AT plates, growth in –Ura plates and *LacZ* expression. Clones that passed all three tests were determined to represent strong interactions. Weak interactions were identified in clones that while growing in –His + 3AT plates and metabolizing Xgal, failed to show detectable growth in Uracil dropout media. The validated interactions between KREV/Rap1A, a member of the Ras family of GTP binding proteins and RalGDS, a guanine dissociation stimulator protein, were used as positive control^33,34^. As weak and negative control, RalGDS mutants (RalGDS-m1 and m2) that differently affect the interaction with Krev were used according to the The ProQuest Two-Hybrid System (Invitrogen). A Diagram of tested plates and with controls transformations is shown on the bottom: pEX32-Krev1 + pEX22-RalGDS-wt (strong interaction); pEX32-Krev1 + pEX22-RalGDS-m1 (weak interaction); pEX32-Krev1 + pEX22-RalGDS-m2 (- interaction); pEX32-*sgo-1* + pDEST22 empty (bait and empty prey) and pEX32 empty + pDEST22 empty (empty bait and empty prey). (**C**, **D**) SGO-1::tdTomato and TAC-1::GFP expressed under the *Posm-5* promoter co-localize in the base of cilia in amphid (C) and phasmid (D) cilia.

**Figure 4:**
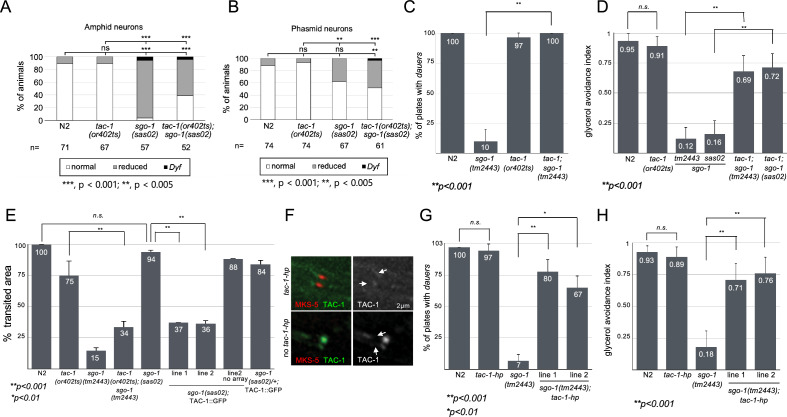
Suppression of *sgo-1* phenotypes by a *tac-1* mutation*.* For all experiments using *tac-1(or402)* escapers, worms tested were raised at 25 °C. (**A**, **B**) Suppression of dye-filling defects quantified in amphid and phasmid cell bodies. Relative signal intensity normalized to N2: 0%, "Dyf"; 1 ~ 66%, ”reduced"; 66 ~ 100% or over 100%, “normal”. (**C**) Suppression of dauer formation (three experiments, 10 plates per genotype). (**D**) Suppression of glycerol avoidance (data represents n = 30 worms for each genotype, with each worm being tested ten times total). (**E**) Suppression of roaming defects. Data is normalized to N2 = 100% and represents four independent experiments with n = 10 for each genotype. (**F**) Knockdown of TAC-1::GFP in worms expressing a *tac-1* mRNA hairpin (*tac-1-hp*) in sensory neurons. Non-transgenic TAC-1::GFP expressing siblings that lost the *tac-1-hp/mks-5::mCherry-*marked array were used as control. Arrows point to the TZ in phasmid cilia. (**G**) Expression of *tac-1-hp* in *sgo-1(tm2443)* animals results in suppression of the roaming (**G**) and glycerol avoidance (**H**) phenotypes.

Given that centromeric Shugoshin acts as an adaptor in the recruitment of effector proteins in the nucleus, we hypothesized that SGO-1 may play a similar role in localizing cilia regulators in *C. elegans*^1^. To search for proteins that interact with SGO-1 in *C. elegans*, we screened a yeast two-hybrid prey library with a full-length SGO-1 bait construct. We identified the tumor overexpressed gene (TOG) domain protein TAC-1, an ortholog of the evolutionarily conserved TACC (transforming acidic coiled-coil) protein family, as an over-represented hit in this screen (> 99% of hits; n = 250) (Fig. 3A). We confirmed this interaction using inverted bait/prey construct assays (Fig. 3B). TAC-1 is a centrosomal-spindle microtubule organizer recently shown to load alongside other pericentriolar proteins in the base of cilia, where these proteins reportedly participate in sensory neuron development^30,31^. We found that TAC-1 co-localizes with DYF-19 in the BB of cilia in sensory neurons but is notably excluded from the TZ and axoneme (Supplementary Figures 7 and 8). Consistent with a physical interaction between TAC-1 and SGO-1 at the base of cilia, both proteins overlap in the BB of amphid and phasmid cilia (Fig. 3C,D). To assess the role of TAC-1 in the BB, we queried for cilia-related phenotypes in *tac-1(or402ts)* mutants. The *or402* mutation affects the evolutionarily conserved TOG domain of TAC-1, disrupting its recruitment to the centrosome on a temperature-sensitive basis. At 25 °C (non-permissive temperature), over 95% of *tac-1(or402ts)* early embryos arrest in anaphase with short astral microtubules, defects in spindle positioning and pro-nuclear extrusion^32^. We addressed whether *tac-1(or402ts)* escapers displayed phenotypes associated with cilia defects when raised for four generations at 25 °C. We found no evidence for this: *tac-1* worms showed regular dye filling, dauer formation, avoidance response to glycerol, and roaming behaviour (Fig. 4A,B,C,D,E). While we cannot rule out residual TAC-1 activity in cilia of *tac-1(or402ts)* worms, these results suggest that TAC-1 depletion per se may not be enough to disrupt cilium biogenesis and function. On the other hand, if SGO-1 and TAC-1 act synergistically in a protein complex at the base of cilia*, tac-1;sgo-1* double mutants may show an enhancement of the cilia defects compared to the *sgo-1* single mutant. Surprisingly, with the exception of dye-filling defects in phasmid neurons, we find instead a significant improvement of most *sgo-1* phenotypes in a *tac-1;sgo-1* genetic background, suggesting that the *tac-1* allele *or402* acts as a suppressor of *sgo-1* mutations (Fig. 4A,B,C,D,E). Given the physical association of SGO-1 and TAC-1, the genetic interaction between *sgo-1* and *tac-1* is consistent with SGO-1 inhibiting TAC-1 at the base of cilia in sensory neurons. In this scenario, unchecked TAC-1 activity in cilia may entirely or partially explain the sensory defects in *sgo-1* mutants, a prediction supported by the enhancement of roaming defects observed when overexpressing TAC-1::GFP in *sgo-1(sas02)* animals to levels comparable to the ones in *tm2443* worms (Fig. 4E). We confirm this possibility by depleting TAC-1 in sensory neurons of *sgo-1(tm2443)* worms expressing a *Posm-5* driven *tac-1* hairpin (*tac-1 hp*) construct (Fig. 4F). As predicted and in agreement with the double mutant analysis, knockdown of *tac-1* specifically in neurons of *sgo-1(tm2443)* animals is sufficient to significantly suppress the dauer formation and glycerol avoidance defects in these mutants (Fig. 4G,H). These results are consistent with a model in which SGO-1 counteracts TAC-1 at the base of the cilia in sensory neurons.

### SGO-1 is required to compartmentalize TAC-1 in cilia

**Figure 5:**
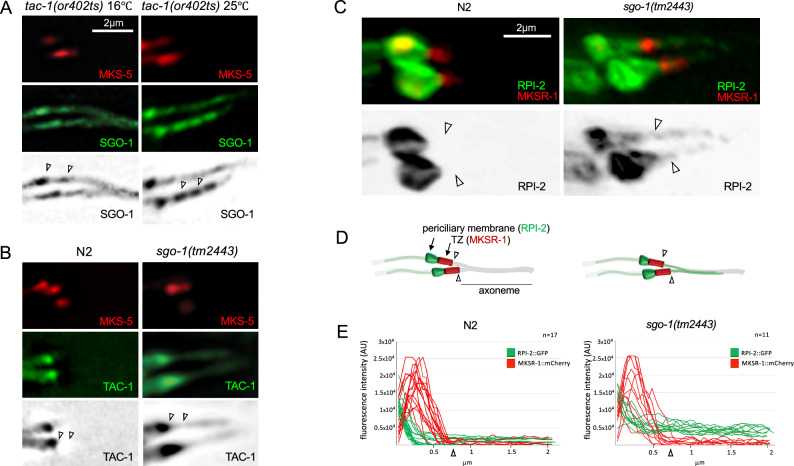
*sgo-1(tm2443)* mutant cilia have gating defects. (**A**) SGO-1::GFP expression in PHA/B cilia of *tac-1(or402)* temperature sensitive mutants raised at the permissive (16 °C) and restrictive (25 °C) temperature. SGO-1 is observed in the base, proximal to MKS-5::mCherry domain, in the TZ (MKS-5 domain) and strongly along the axoneme (distal to MKS-5 domain). (**B**) TAC-1::GFP expression in PHA/B cilia of N2 or *sgo-1(tm2443)* worms. TAC-1 is restricted to the base of cilia (proximal to MKS-5 domain) and not observed in the TZ (MKS-5 domain) or axoneme (distal to MKS-5 domain) in N2 worms while in *sgo-1* mutants, TAC-1 spreads into the TZ as well as along the axoneme. Inverted black and white images of the GFP panes are shown on the bottom. The TZ boundaries are indicated between the arrowheads. (**C**) Diffusion of RPI-2::GFP into the TZ and axonemal domains of phasmid cilia of N2 and *sgo-1(tm2443)* worms. Inverted black and white images of RPI-2::GFP are shown on the bottom. The TZ/axoneme distal boundary in each cilium is indicated by arrowheads. (**D**) Diagrams of phasmid cilia showing RPI-2 and MSKR-1 domains as observed in N2 (left) and *sgo-1(tm2443)* (right) worms. Arrowheads mark the TZ/Axoneme boundary. (**E**) Quantification of MKSR-1::mCherry and RPI-2::GFP fluorescence signals along a 2 mm transect from the proximal TZ region (marked by MKSR-1 signal) distally through the TZ and axonemal domains.

How does SGO-1 inhibit TAC-1 in cilia? Shugoshins are known to transiently bind pericentromeric domains to spatially and temporally regulate the access of effector enzymes to substrates^1^. Therefore, we considered whether SGO-1 regulates TAC-1 localization within the basal body-cilia organelle, a highly compartmentalized cellular structure. We tested this hypothesis by investigating the interdependencies in the ciliary localization of TAC-1 and SGO-1 in their respective mutant backgrounds. We did not notice changes in the localization of SGO-1 in cilia of *tac-1(or402ts)* animals (Fig. 5A). Conversely, whereas TAC-1 was restricted to the BB of N2 cilia, TAC-1::GFP signal can be observed in distal ciliary domains in *sgo-1(tm2443)* mutants, including the TZ and more distal regions of the axoneme (Fig. 5B; Supplementary Figure 8A,B,C,D).

If free TAC-1 is restricted proximally by titration with N2 SGO-1, depletion of SGO-1 should also result in passive diffusion of TAC-1 into cilia. Indeed, TAC-1::GFP diffuses into ciliary axonemes in *sgo-1(sas02)* mutants (Supplementary Figure 9A,B). We reasoned that the mis-localization of TAC-1 into the cilia of *sgo-1* mutants might have functional consequences and underlie some of the ciliary defects observed in these animals. Accordingly, *sgo-1(sas02)* worms expressing TAC-1 transgenes have roaming defects reminiscent of those observed in *sgo-1(tm2443)* worms (Supplementary Figure 4E). The roaming defect and cilia mis-localization of TAC-1 appear to be triggered by added TAC-1 levels in *sas02* homozygotes expressing TAC-1::GFP, since *sas02* animals with endogenous TAC-1 levels are not dwellers (Fig. 2F) nor are *sgo-1(sas02)/* + heterozygotes expressing the TAC-1 transgene (Fig. 4E). We concluded that decreased SGO-1 function pre-sensitizes cilia to TAC-1, which then enters cilia unchecked. These findings support a balance between SGO-1 and TAC-1 activities within the ciliary base to ensure proper cilia structure and/or function. If this hypothesis is correct, it should be possible to dominantly induce the sensory defects observed in *sgo-1* mutants by altering the SGO-1/TAC-1 ratio in N2 neurons. We found that N2 animals overexpressing transgenic arrays produced with 10 × higher concentrations of *tac-1::gfp* transgenes show an abnormal localization of TAC-1 within cilia, and phenocopied sensory defects observed in *sgo-1* mutants (Supplementary Figure 9C,D,E). These observations lead us to propose that occupancy of the TZ and axoneme by TAC-1 disrupts cilia biogenesis and/or function, and that this outcome is normally prevented by SGO-1, which promotes cilia function not by recruiting TAC-1 but rather by spatially limiting the TAC-1 domain to the ciliary base.

In the early *C. elegans* embryo, TAC-1 ensures proper spindle assembly by promoting microtubule growth radiating from the centrosome^35^. Conversely, mammalian SGOL2 inhibits incorrect kinetochore/spindle attachments by mediating microtubule depolymerization via MCAK recruitment to the centromere^36^. Considering this antagonistic relationship of TACC and SGO proteins in regulating microtubule growth during cell division, we hypothesized that the balance of SGO-1 and TAC-1 activities at the base of cilia may ultimately regulate axonemal microtubule stability, potentially impacting axonemal length. To test this, we probed for changes in cilium length using an established ciliary marker expressed in ADL neurons^37^. The *sgo-1(sas02*), *sgo-1(tm2443)*, and *tac-1(or402ts)* mutants did not, however, show any significant differences as compared to N2 cilia (Supplementary Figure 10A,B). These results suggest a mechanism for TACC and Shugoshin in cilia likely distinct from their counterpart roles in other microtubule-based structures in the cell.

### *sgo-1(tm2443)* mutants have ciliary gating defects

The transition zone (TZ) functions as a diffusion barrier that is largely responsible for the compartmentalization of the ciliary organelle. This ‘ciliary gate’ selectively sorts molecules crossing in and out of cilia, fundamentally changing their concentrations in different cilia regions^38–40^. Considering SGO-1’s localization to the TZ and the abnormal diffusion of TAC-1::GFP into the TZ and distal ciliary regions in *sgo-1* mutants, we investigated whether SGO-1 plays a more general role in supporting the diffusion barrier. We used animals expressing the worm ortholog of retinitis pigmentosa 2, RPI-2, fused to GFP for these assays. In wild type animals, RPI-2::GFP accumulates in the periciliary membrane (near the basal body) of sensory neurons, showing little to no signal within the ciliary axoneme^40^. However, when proteins that localize specifically to the transition zone are disrupted in *C. elegans*, including MKSR-1, MKSR-2, MKS-5, MKS-6 and CEP-290, RPI-2 is found to accumulate within the axoneme, revealing a ‘gating’ defects^41,42^ A similar gating phenotype is observed with transition zone mutants when a different periciliary membrane marker, TRAM-1, is used^39,43,44^. In contrast to N2 and *sas02* mutants, 88% (n = 50) of phasmid cilia in *sgo-1(tm2443)* worms showed RPI-2::GFP crossing the TZ and into the axonemal domain (Fig. 5C,D, quantified in Fig. 5E; Supplementary Figure 10C, quantified in Supplementary Figure 10D). The magnitude of RPI-2 accumulation in the various TZ mutants and the *sgo-1* mutant is somewhat variable, but clearly different compared to wild type. Importantly, this ectopic localization of RPI-2::GFP is detectable in a SGO-1^*tm2443*^/SGO-1^WT^ dose-dependent way in phasmid cilia of *tm2443/* + (30%; n = 40) and *tm2443/sas02* (44%; n = 54) animals and can be further induced in a N2 *sgo-1(* +*)* background over expressing SGO-1^*tm2443*^ (58%; n = 45) (Supplementary Figure 10D). In concert, these findings raise the possibility that in addition to its role in the BB, SGO-1 may also act in the TZ to sustain its diffusion barrier functions and regulate the composition of the ciliary compartment.

## Discussion

The presence of the *C. elegans* Shugoshin homolog in post-differentiated neurons points to a novel function for this family of cell cycle-associated proteins. Previous studies in mice identified *Sgo1* in subsets of developing and post-developmental retinal cells^10^, while transcriptomic analyses reported enrichment of *sgo-1* transcripts in the amphid sensory organ of *C. elegans*^22,23^. Thus, the expression of Shugoshin genes in post-differentiated somatic tissues is likely a conserved feature of this gene family. Our study expands these findings by identifying a Shugoshin-binding protein, the microtubule regulator TAC-1, and functionally implicating both in cilia function and sensory perception in neurons.

SGO-1’s localization within the basal body-ciliary compartment of sensory neurons was as unexpected as it was intriguing, considering the reported roles of Shugoshin proteins at the centrosome, spindle, and kinetochore, the other microtubule organizing centers in the cell^45–47^. Shugoshin spatially and temporally limits unregulated microtubule polymerization and promotes depolymerization in these contexts. Similarly, a correct balance of microtubule polymerization and depolymerization in cilia is critical for establishing a functional axoneme with a specific length^48^. For instance, depletion of the TZ protein KLP-7, a depolymerizing kinesin that also controls microtubule dynamics in the centrosome and kinetochore, results in shortened axonemes and defective cilia^37,49^. Some components of the conserved KMN (Knl1/Mis12/Ndc80-complex) network, involved in kinetochore-microtubule-coupling during mitosis, also function to promote the extension of developing dendrites in *C. elegans*^50^. The presence of these modules in dendrites draws a functional link between these two processes that are heavily reliant on the microtubule cytoskeleton and suggest that the machinery involved in microtubule-dependent events in the nucleus has been co-opted to sustain sensory neuron morphogenesis.

Controlling microtubule polymerization and axonemal growth/length would seem a logical target for Shugoshin activity in cilia. Consistent with this possibility, we found that SGO-1 physically interacts with TAC-1, a TOG domain containing MAP (microtubule-associated protein) essential for promoting centrosomal and spindle microtubule growth in *C. elegans*. Importantly, *C. elegans* neurons in *tac-1* mutants assemble shorter microtubule bundles, a feature associated with the disrupted axonal microtubular organization and cargo trafficking in these cells^51^. Moreover, TAC-1 and other centrosomal maturation proteins in the pericentriolar material have been recently shown to occupy the base of cilia in worms, a domain unique in *C. elegans* that lacks intact centrioles but retains γ-tubulin^30^. Our epistasis analysis indicates that TAC-1 is the effector of SGO-1 activity in sensory neurons, as defects in *sgo-1* mutants can be explained by upregulation of TAC-1 function in cilia. However, we found no direct evidence of defects in axonemal length in *sgo-1* or *tac-1* mutants. Whether and how SGO-1 and TAC-1 may influence microtubule dynamics in cilia is therefore still unresolved but worthy of further investigation.

Our results are consistent with a model in which SGO-1 promotes cilia function by downregulating TAC-1 activity. What is the mechanism behind SGO-1 inhibition of TAC-1 in the base of cilia? Shugoshin’s adaptor roles in the centromere and kinetochore are exerted by the transient recruitment of kinases and phosphatases to its sites of action in the cell. In the centrosome, vertebrate TACC is phosphorylated by Aurora-A to engage XMAP215-dependent microtubule polymerization^52^. Similarly, the *C. elegans* AIR-1 has key roles in mitotic centrosome assembly, but we and others failed to detect it in worm cilia^30^. Considering that SGO-1 directly binds TAC-1, presumably within the base of cilia where these proteins co-localize but is not required for TAC-1 recruitment to this domain, we explored the possibility that SGO-1 regulates TAC-1 by spatially limiting its cilia occupancy. Indeed, we find that TAC-1 spreads to distal cilia domains (TZ and more distal axonemal regions) in *sgo-1* mutants, suggesting that ectopic TAC-1 activity in the TZ and/or axoneme is behind the sensory defects observed in these worms. Consistent with this interpretation, overexpression of TAC-1 in N2 animals with normal SGO-1 function results in TAC-1 ectopic localization to the TZ and distal cilia domains, and phenocopies sensory defects observed in *sgo-1* mutants.

**Figure 6:**
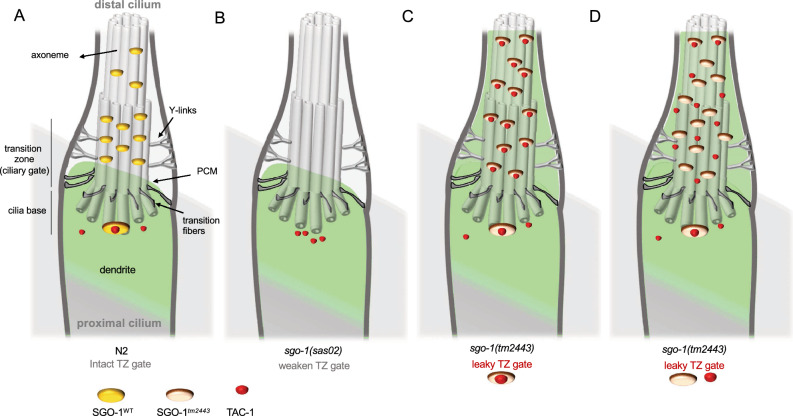
(**A**) In wild type (N2) worms, SGO-1 binds to TAC-1 in the base of cilia. SGO-1 is also observed along the TZ and axoneme, both TAC-1-free domains in wild type cilia. In the TZ, activity of SGO-1 is likely important to sustain the impermeability of the ciliary gate (green). (**B**) Depletion of SGO-1 disrupts ciliary function, as attested by the behavioral defects in *sgo-1(sas02)* worms. While we did not observe gating defects in cilia of *sas02* worms, increasing TAC-1 in these cilia (but not cilia of *sas02/* + worms), disrupts the diffusion barrier. TAC-1 is widely distributed beyond the TZ gate in both *sas02* and *tm2443* worms, though sensory defects in the latter are markedly more severe. We interpreted these results as indicative that SGO-1-less cilia have a weakened gate, predisposed to loss of integrity, while SGO-1^*tm2443*^ enhances cilia dysfunction by enhancing TAC-1 activity in distal cilia domains of *tm2443* worms. This effect of SGO-1^*tm2443*^ could be accomplished by binding and stabilizing TAC-1 in these domains (**C**) Conversely, TAC-1 may passively diffuse across the disrupted TZ of *tm2443* mutants and remain unbound to SGO-1 in distal domains (**D**). PCM—periciliary membrane.

Notably, we also detected diffusion of RPI-2::GFP, a marker for ciliary gate integrity, from the periciliary region into cilia in worms with defects in SGO-1. This is reminiscent of a more general gating defect often observed in mutants with disrupted TZ structure^53^. The genetic analysis of *sgo-1(sas02)* and *sgo-1(tm2443)* alleles strongly supports the view that sufficient SGO-1 function in cilia is required to restrain TAC-1 activity (Fig. 6). Depletion of SGO-1 in *sgo-1(sas02)* worms perturbs this equilibrium and introduction of ectopic TAC-1 to the system significantly worsens sensory outcomes. The more prominent phenotypes in *sgo-1(tm2443)* worms are also paralleled by TAC-1 mis-localization but differently than *sas02* worms, they respond to a dominant gain of function role of SGO-1^*tm2443*^ in cilia. Further understanding of the function of the *tm2443* domain in the wild type SGO-1 protein may help clarify the mechanism behind the SGO-1^*tm2443*^ -mediated cilia phenotypes. Finally, while our results implicate TAC-1 in the sensory defects uncovered in *sgo-1* mutants, the presence of SGO-1 in ciliary regions lacking TAC-1 suggests TAC-1-independent roles of SGO-1 at the TZ and within the axoneme. We anticipate that the identification of novel binding targets of SGO-1 in worms will contribute to dissecting these functions. Together, this work points to a broad role of SGO-1 in supporting cilia compartmentalization and homeostasis via, at least in part, TAC-1 regulation, and a role in supporting the function of the TZ ‘ciliary gate’.

## Methods

### *Caenorhabditis elegans* genetics and transgene generation

Worms were maintained on NGM plates at standard growth conditions^54^. The lists of strains, plasmids and primers used in this study are available in the Supplemental Material. To test *tac-1(or402ts)* temperature sensitive mutants, escapers were grown at 25 °C for at least three generations. L4 escapers were selected from plates that had been maintained at 25 °C for at least 5 days before the assay. Standard genetic crosses and PCR-based genotyping of *tm2443* and *sas02* were used to generate and monitor mutant lines. Stably transmitting transgenic worms were generated by microinjecting 15 μg/ml of DNA constructs with 100 ng/ml of PvuII-digested *E.coli* genomic DNA. For overexpression of *tac-1* in *sasEx44* and *sasEx45*, 100 ng/ml of pCEC41 was used. Co-injection markers used were: pRF4 [*rol-6(su1006)*]; pMM05 [*unc-119(* +*)*]; *Podr-1::DsRed* and ccGFP (*Punc-122::GFP*). Transgenic lines were maintained as chromosomal arrays. sasIs05 is a spontaneous integration. *Psgo-1::GFP* transcriptional reporter in *sasEx01* was made by fusion PCR. Primers prCC1/2 were used to amplify a 990 bp promoter fragment that was fused to a GFP cassette amplified from pPD95.79 using prCC3/4. For rescue experiments, a 3.3 kb *sgo-1(* +*)* genomic fragment was PCR amplified from N2 gDNA using primers CC116F/R and cloned into pSC-B (Agilent) to generate pCEC02. This fragment includes ~ 1.6 kb upstream of the first ATG and ~ 380 bp of *sgo-1* 3’UTR. To drive expression in ciliated neurons, 323 bp of the *osm-5* promoter containing the DAF-19 binding X-box sequence was used^55^. To generate a *tac-1* mRNA hairpin, a 563 bp fragment of *tac-1* cDNA encompassing exon 1 and exon 2 was cloned upstream of a 373 bp GFP spacer sequence followed by the reverse complement *tac-1* sequence to generate pCEC54. *tac-1* mRNA knockdown was verified by assessing the relative drop in TAC-1::GFP signal in N2 worms co-expressing *sasEx57* and *sasEx66*. To express SGO-1^*tm2443*^ in sensory neurons, the *sgo-1* genomic sequence lacking the 195 bp deletion in *sgo-1(tm2443)* worms was fused in frame with a mCherry cassette downstream of *Posm-5* (pCEC56). All constructs carry a unc-54 3’UTR unless specified. p328.1 and p330.1 were gifts from the B.K Yoder^56^. The *mks-5/dyf-19::mCherry* plasmids were provided by J. Hu^57^. Strains were obtained from *the Caenorhabditis Genetics Center* (CGC).

### Microscopy

Worms were anesthetized on 25 mM of sodium azide in M9 buffer and mounted on 2% agarose pads for live imaging. Images are projections of 3D stacks collected using a Delta Vision (GE) deconvolution system (0.2 μm optical sections) or a WaveFX spinning disc confocal microscope (Quorum Technologies). Deconvolution, when necessary, was performed using Softworxs (GE). Expansion of TAC-1::GFP domain into the TZ and axoneme was quantified using the line profile tool in Softworks. Relative fluorescence intensities collected from phasmid cilia using the same acquisition parameters were measured in N2 (n = 10) and *sgo-1(tm2443)* (n = 14) worms expressing MKS-5::mCherry and TAC-1::GFP across a 2 mm transect traced from the base of the TZ (boundary between the MKS-5::mCherry (TZ) and TAC-1::GFP (BB) domains) along the axoneme distally. Images of worms expressing *Pcol-183::mCherry* were taken on a Ds-Fi1 camera mounted on a Nikon SMZ1500 fluorescent stereomicroscope.

### *tm2443* sequencing

cDNA was produced from N2 and *sgo-1(tm2443)* worms using Trizol-extracted RNA (Invitrogen) and oligo dT primers. The *sgo-1* locus was amplified with Phusion Taq using prCC83 (exon1) and prCC84 (exon7) primers. Adenine overhangs were added using Taq polymerase and fragments T-A cloned into pGEM-T (Promega) to make pCEC11. Sequence was performed using T7 and SP6 primers. For genomic analysis, N2 and *sgo-1(tm2443)* genomic DNA were amplified with prCC88 and prCC84, cloned in blunt into pSC-B and sequenced with T3 and T7 primers.

### DiI assays

Dye-filling assays were carried out using 1:1000 dilution DiI solution (Molecular Probes or Invitrogen) in M9 buffer^58^. Worms from mixed populations were washed off plates with M9 and stained for 30 min in 500 μl of Dil solution. Before imaging with fluorescence microscopy, worms were washed twice in M9 buffer and placed on food plates for 1 h to clear excess DiI. Dye filling phenotypes in phasmid neurons were classified in four groups (1) WT: continuous staining of all dendrites and even presence of dye signal in the cell bodies of all 4 phasmid neuron (PHAR, PHAL, PHBR, PHBL); (2) Dyf: complete lack of staining in dendrites or neuronal cell bodies; (3) Dyf: incomplete staining in at least one of the 4 cells bodies; (4) Dyf: patchy (foci) or weak staining along dendrites of at least one phasmid neuron. Immediately after staining and washing, 40 worms per genotype were mounted on glass slides and imaged in a Delta Vision Elite microscope. Experiments were repeated twice for each genotype. Worms classified in groups 2, 3 and 4 were pooled together to calculate the % of animals displaying dye filling defects. For quantification of dye-filling defect suppression, the signal intensity of fluorescent dyes found in amphid and phasmid cell bodies was obtained using ImageJ software. Signal intensity observed in the mutant strains was normalized to the N2 average signal.

### Chemotaxis assays

Avoidance behaviour was quantified using the drop test method^59^. Briefly, about 5 μl of freshly made repellent solutions (3 M glycerol) in M13 buffer was dispensed near the tail of a forward moving adult worm. A backward movement response within 4 s of contact with the solution was scored as avoidance response. N2 and *osm-6(p811)* worms were used as positive and negative controls, respectively. Tested worms were singled on foodless plates at room temperature and allowed to roam for 10 min before tests began. A lack of response of N2 worms exposed to M13 buffer was confirmed before each experiment. Experiments consisted of 30 worms per genotype with each worm being tested 10 × with intervals of at least 2 min between tests. Avoidance indexes (AI) for each worm were calculated as the number of positive avoidance movements divided by the number of tests and AI averages derived. Only worms that completed all 10 tests were considered for calculation.

### Roaming assay

Tracking was assessed as described^26^. A single adult worm was allowed to roam on a standard (3 cm^2^) OP50 lawn for 16 h at 20 °C after which the worm was removed and presence of tracks on 100 3mm^2^ square grid scored. Four independent experiments with at least 10 worms each were performed for each data set. N2 controls tested in each experiment were used to normalize the data.

### Dauer formation assay

Dauer formation was assessed using SDS resistance assays^60^. In short, worms were grown on standard (1 cm^2^) OP50 lawns at 20 °C or 25 °C (for *tac-1* temperature sensitive mutants) until starvation. 5 days after starvation worms were collected and washed in M9, resuspended in 1 ml of 1% SDS and incubated with gentle shaking for 30 min. The 1 ml solution was dispensed on a plate lid and the presence or absence of thrashing dauer worms was scored under a stereoscope. Three experiments, each composed of 10 plates per genotype, were carried out. The % average of plates with dauers in the three experiments was used. For dauer entry analysis, fluorescence in post-starvation worms expressing *Pcol-183::mCherry (syIs600)* worms was followed^61^. For quantification of dauer formation, worms were washed independently from 5 plates at 72 h post-starvation and resuspended in 200 μl of M9. 10 μl of 20 mM Sodium Azide was added to 15 μl of resuspended worm solution and mounted on glass slides. Experiments were repeated twice. Scoring of the number of worms with mCherry signal was carried out under a Nikon SMZ1500 stereomicroscope.

### Axonemal length measurements in ADL neurons

L4 larval N2 and mutant worms expressing IFT-20::GFP were used for the ADL ciliary length measurement (excluding basal body). All images were analyzed with the Volocity software, and the data were plotted using Dot and Boxplots in R software. The Shapiro–Wilk test determined the data distribution, and *p* values were calculated by the Dunn’s Kruskal–Wallis Multiple Comparisons (Holm-Sidak adjustment).

### Yeast two-hybrid screen

A full *sgo-1* cDNA fragment was generated using primers B1 and B2 and Superscript II Reverse Transcriptase (Invitrogen). The B1 forward primers contains a yeast Kozak consensus to aid expression of the bait construct. The *sgo-1* cDNA insert was cloned into pDONR-221 to generate pENTR-2210-*sgo-1* and sequence-checked with primers prCC332 and M-13. Gateway cloning was used to generate the expression vector pExp-32-*sgo-1*. Yeast Two-Hybrid screening and selection assays were performed using ProQuest Two-Hybrid System (Invitrogen), according to the manufacturer’s protocol. In short, a *C. elegans* prey cDNA library in pDEST-22 (a gift from S. Smolikove) was co-transformed in MaV203 cells with the pExp-32-*sgo-1* bait vector. Approximately 5.78 × 10^6^ individual double transformant colonies were screened on HIS- URA- plates. A concentration of 10 mM 3AT was found to be sufficient to supress self-activation of the bait vector. Plasmid DNA from positive hits was isolated, transformed into electrocompetent DH5a cells and recovered for sequencing using primers prCC333 and prCC335. SGO-1 binding of selected hits identified through sequencing were subsequently confirmed in Lac Z reporter assays.

### Statistics

Average results were calculated for each experiment replicate. Using the averages from each replicate, *p* values were calculated in two-tailed *t* tests with equal variance in Excel. For suppression of dye-filling defects, Dunn's Kruskal–Wallis test: Holm-Sidak was used for amphid data, and Tukey HSD was used for phasmid data.

## Supplementary Information

Supplementary Information 1.

Supplementary Information 2.

Supplementary Information 3.

## Data Availability

All data used in this manuscript is in the Open Science Framework database at: https://osf.io/h35tm/. *tm2443* sequencing data is available at GenBank under the following accession numbers: OQ024003-OQ024005.
