# Supplementary material for: The Caenorhabditis elegans Shugoshin regulates TAC-1 in cilia
Source: Sci Rep. 2023 Jun 9;13:9410. doi: 10.1038/s41598-023-36430-8 (PMC10256747; doi:10.1038/s41598-023-36430-8)
Supplement: Supplementary file 2 — Supplementary Information 2. [file 41598_2023_36430_MOESM2_ESM.pdf]

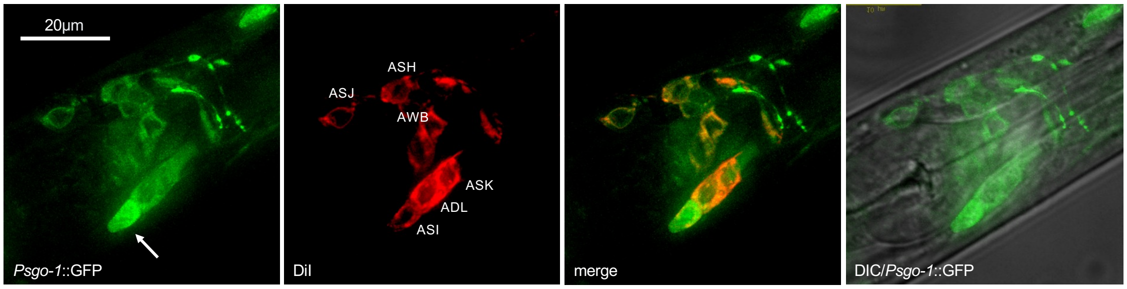

Figure S1

FIGURE S1 – *sgo-1* expression in amphid neurons. A *sgo-1* transcriptional reporter (*Psgo-1::GFP*) carrying 900 bp of 5' sequences fused to GFP (green) is expressed in all 6 pairs of DiI-stained sensory neurons (red) with particularly high expression observed in ASI neurons (arrow). Expression is not exclusive of sensory neurons (data not shown), suggesting that SGO-1's roles in the nervous system are not limited to cilia.

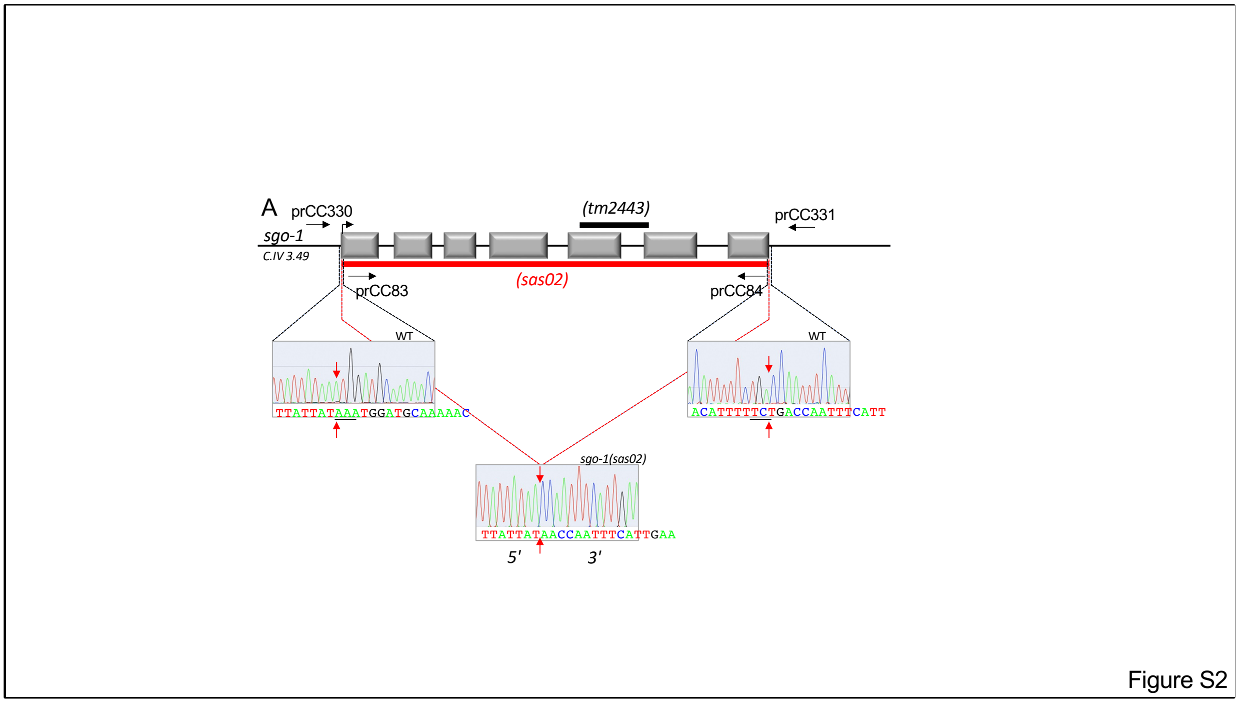

FIGURE S2 – *sgo-1(sas02)* deletion. (A) Diagram of the *sgo-1* locus showing the *sas02* deletion (red bar) and associated sequences. Red arrows indicate the *sas02* deletion boundaries. The deletion in *tm2443* is shown as black bars and primers used for genotyping as arrows.

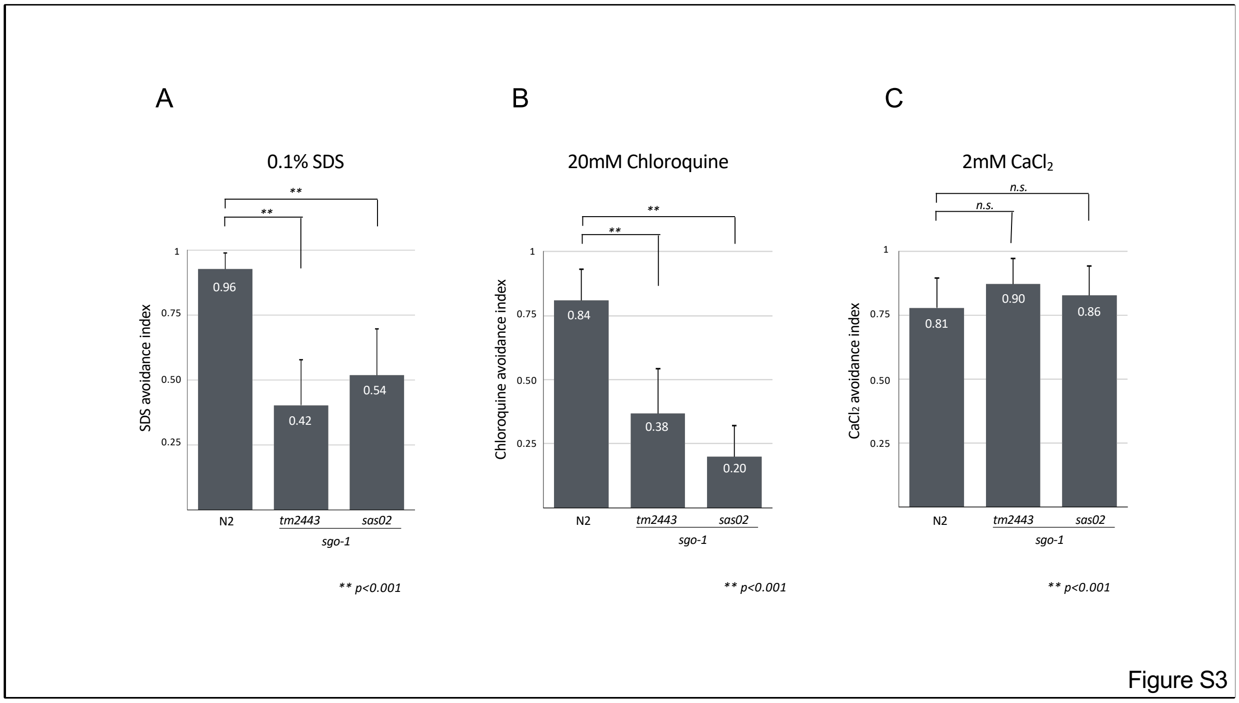

FIGURE S3. Chemical avoidance assays. *tm2443* and *sas02* worms are deficient in SDS (A) and chloroquine (B) avoidance but respond normally with respect to $CaCl_2$ avoidance (C). In all cases, data represents n = 30 animals for each genotype that were tested individually ten times each. n.s., not significant.

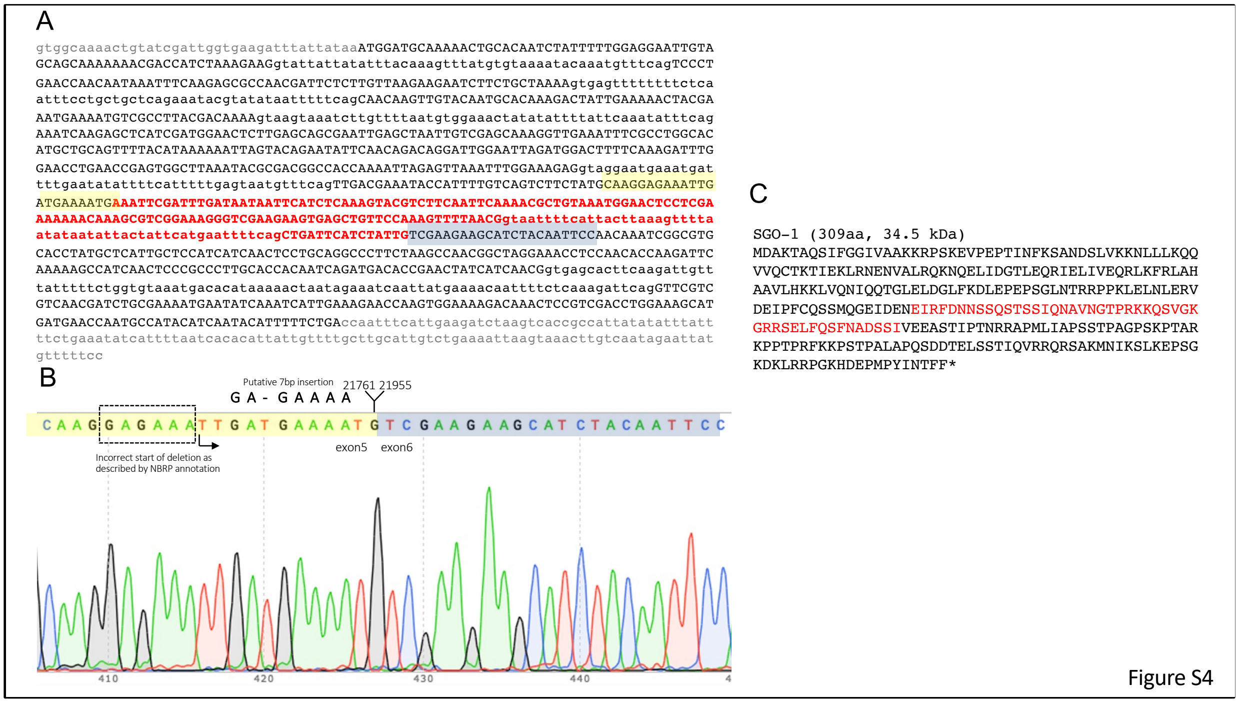

FIGURE S4 – Sequence analysis of *tm2443*. (A) *sgo-1(tm2443)* genomic sequence. The inframe 195bp deletion is shown in red. The deletion spans exons 5, intron 5 and exon 6 sequences. The regions flanking the deletion are highlighted in yellow and blue. (B) Sequenced *tm2443* cDNA showing the deletion boundary region between exon 5/6 (highlighted in yellow and blue, respectively). The previously annotated start of the 204bp *tm2443* deletion is boxed and marked by an arrow. The presumed insertion of 7bp (GAGAAAA, indicated on top line) is likely an annotation mistake and corresponds instead to exon 5 sequences (see end of yellow highlighted). (C) SGO-1 protein sequence. Conceptual translation of *sgo-1(tm2443)* cDNA predicts an inframe SGO-1 protein lacking 47 residues (red sequence) but retaining the N and C termini domains.

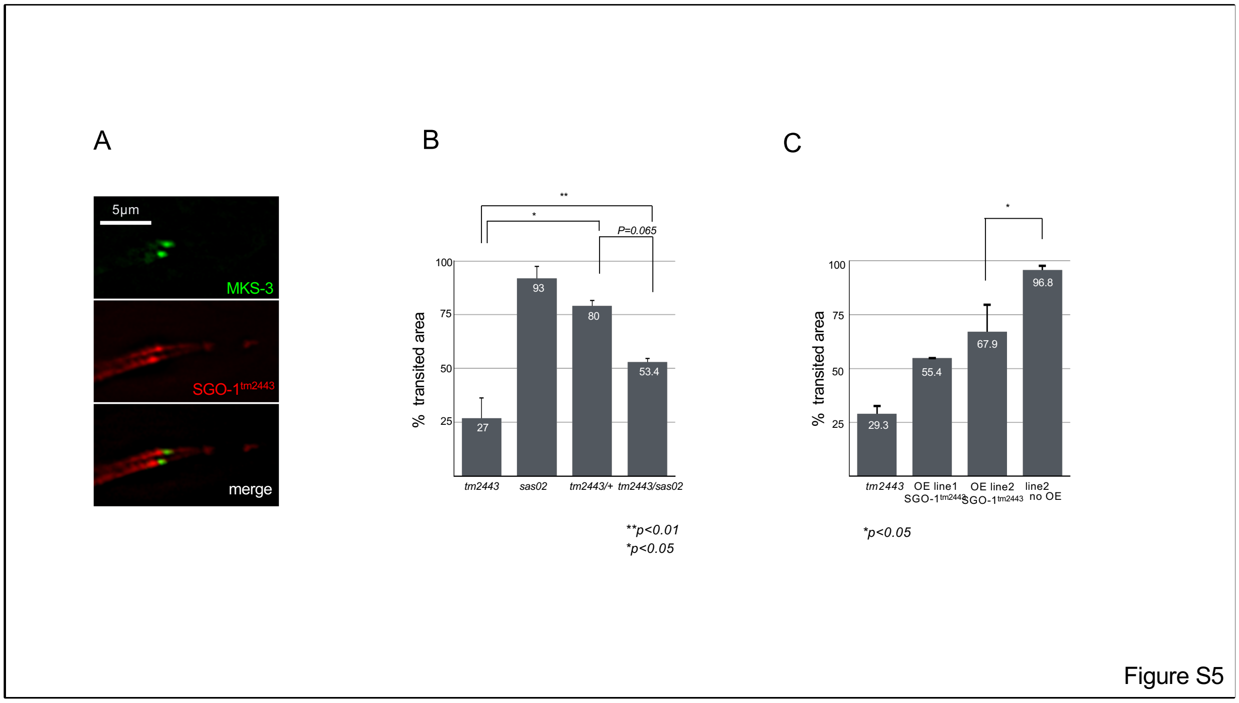

FIGURE S5. *tm2443* is a dose dependent, dominant allele of sgo-1. (A) Expression of a *Posm-5::sgo-1*^{tm2443}*::mCherry* transgene in phasmid cilia. The SGO-1^{tm2443} mutant protein localizes to BB, TZ and axoneme. TZ is marked by MKS-3::GFP. (B) The roaming defect observed in *tm2443* homozygotes is absent in null sgo-1 mutants *(sas02)*. *tm2443/+* heterozygotes show an intermediate level of roaming compared to N2 and *tm2443/sas02* trans-heterozygotes. Data shown is normalized to N2 = 100 and represents four independent experiments with n = 10 for each genotype (C) Roaming assay of lines overexpressing SGO-1::^{tm2443} in sensory neurons (OE = overexpressed). Siblings of OE line 2 were tested as controls. Data shown is normalized to N2 = 100 and represents four independent experiments with n = 10 for each genotype.

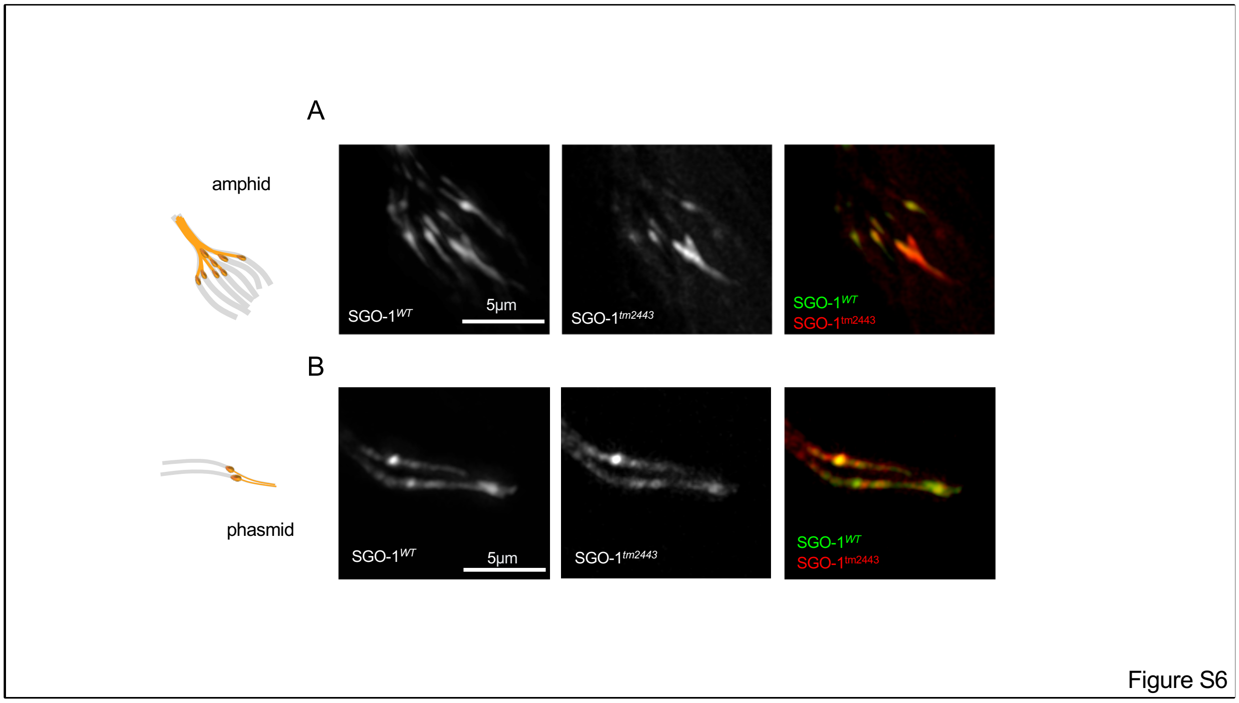

FIGURE S6 – Localization of SGO-1*tm2443* protein in cilia. SGO-1*tm2443* ::mCherry co-localizes with SGO-1::GFP in the basal body, transition zone and axoneme domains when expressed in sensory neurons of head (A) and tail (B).

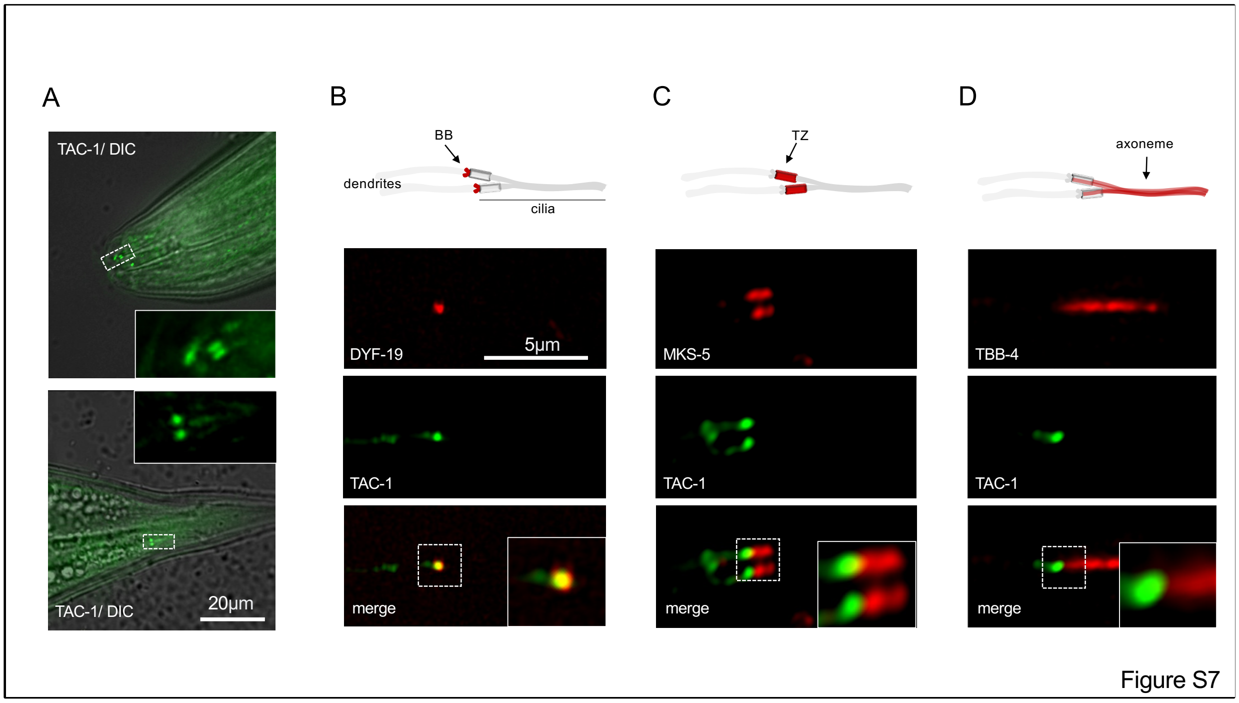

FIGURE S7 – TAC-1 localization to cilia. (A) TAC-1::GFP genomic construct under its endogenous regulatory sequences drives expression of TAC-1 in distinct foci in head and tail, consistent with the positions amphid and phasmid regions (insets). Phasmid images of worms expressing *Posm-5::tac-1::GFP* and *either Parl-13::dyf-19::mCherry* to mark the basal body [BB] (B), *Parl-13::mks-5::mCherry*, marking the transition zone [TZ] (C) or *Posm-5::tbb-4::mCherry* to mark the axoneme (D).

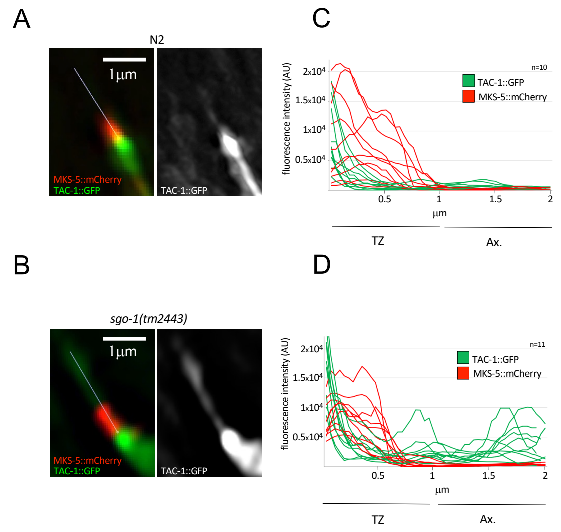

Figure S8

FIGURE S8 – TAC-1 expanded localization to distal cilia in sgo-1(tm2443) worms. TAC-1::GFP signal is observed beyond the basal body domain, into the transition zone (marked by MKS-5::mCherry) and along the axoneme in phasmid cilia of *tm2443* mutants (A,B; quantified in C,D). TZ, transition zone; Ax, axoneme.

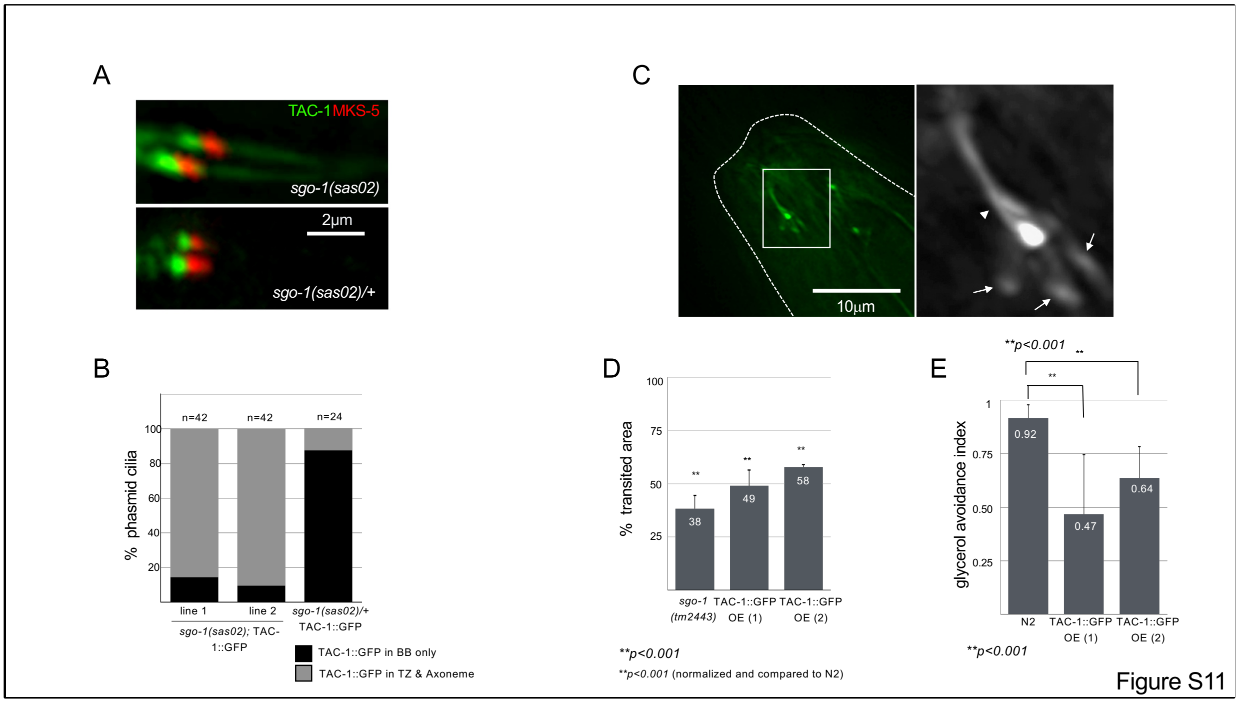

FIGURE S9 – TAC-1 overexpression drives *tm2443*-like cilia defects in *sas02* and wild type animals. (A-B) Diffusion of TAC-1::GFP distally in phasmid cilia of *sgo-1(sas02),* but not *sas02*/+ worms. Representative images are shown in (A) and quantification of the defect in (B). The TZ domain is marked by MKS-5::mCherry. (C,D,E) Overexpression of TAC-1::GFP in amphid cilia of wild type worms can also result in TAC-1::GFP leakage to distal cilia domains and defects in roaming. A representative image of an amphid of worms overexpressing TAC-1::GFP and its diffusion to distal cilia domains is shown in (C). Arrows and arrowhead indicate BB and axonemal regions with TAC-1::GFP signal, respectively. Both roaming defects (quantified in D) and avoidance behaviour (quantified in E) observed in *sas02* homozygotes expressing TAC-1::GFP are reminiscent of those observed in *tm2443* worms. Roaming data shown is normalized to N2 = 100% and represents four independent experiments with n = 10 for each genotype. Glycerol avoidance data represents n = 30 worms for each genotype, with each worm being tested ten times total.

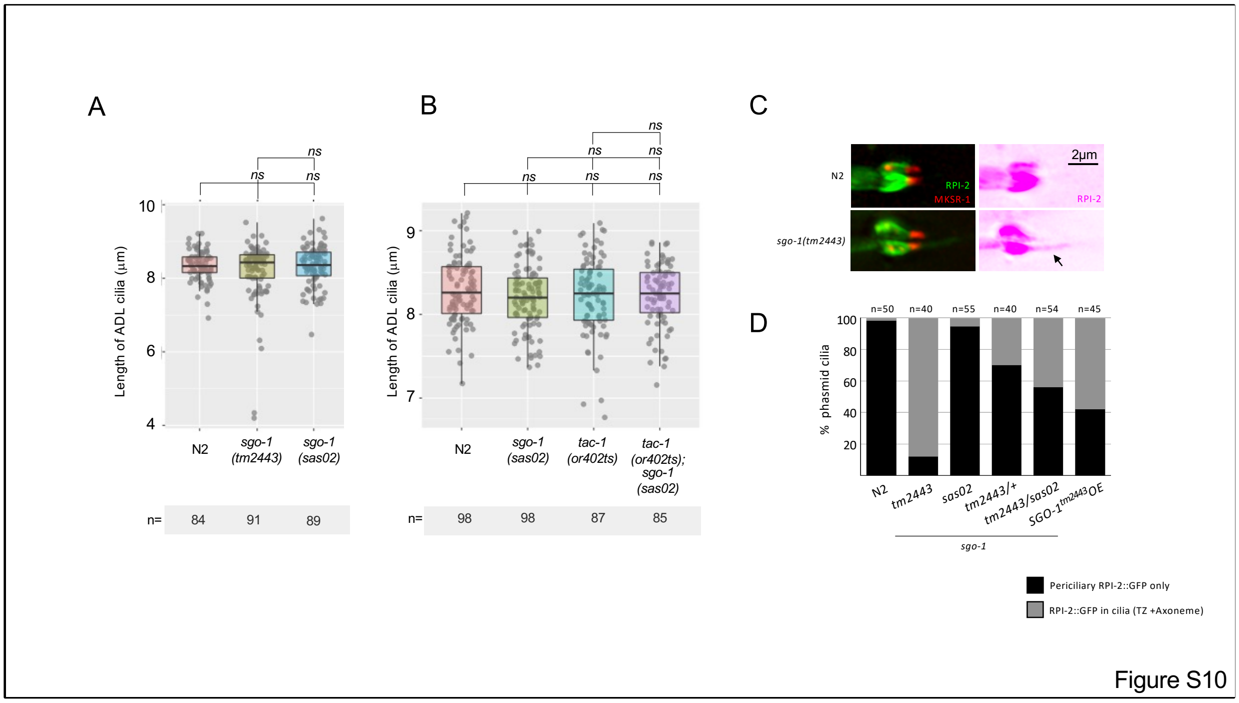

FIGURE S10. SGO-1 and TAC-1 are dispensable for ciliary length regulation, but 'gating' defects are present in *sgo-1* mutants. (A and B) Cilia length measurement in ADL neurons using GFP-tagged IFT-20 in wild type, *sgo-1* and *tac-1* mutants (L4 larvae). One dot represents one cilium. *n.s.*, not significant. (C) Representative phasmid images of wild-type (top) and *sgo-1(tm2443)* worms (bottom) expressing the cilia gate reporter RPI-2::GFP and the TZ marker MKSR-1::tdTomato, arrow points to RPI-2 diffused into the axonemal domain. Right panels show the inverted colour images for RPI2::GFP. (D) Quantification of gating defects.
