# Supplementary material for: The Caenorhabditis elegans Shugoshin regulates TAC-1 in cilia
Source: Sci Rep. 2023 Jun 9;13:9410. doi: 10.1038/s41598-023-36430-8 (PMC10256747; doi:10.1038/s41598-023-36430-8)
Supplement: Supplementary file 3 — Supplementary Information 3. [file 41598_2023_36430_MOESM3_ESM.pdf]

Sup Table 1 – Strains used in this study

**Genotype**	**Paper data**	**Source**	**Strain name**
wild type isolate	-	CGC	N2
sasEx01 [Psgo-1::GFP; rol-6(su1006)]	Fig1, SupFig1	This work	CEC09
sasEx78 [Posm-5::sgo-1::GFP; Parl-13::mks-5::mCherry; ccGFP]	Fig1	This work	CEC286
sasEx79 [Posm-5::sgo-1::GFP; Parl-13::dyf-19::mCherry; ccGFP]	Fig1	This work	CEC287
che-11(tm3433)V; unc-119(ed3)III; vuaSi24[pBP43; Pche-11::che-11::mCherry; cb-unc-119(+)]II; nxEx3103[Posm-5::sgo-1::gfp; rol-6(su1006)]	Fig1	This work	MX3103
sgo-1(tm2443)IV	Fig2	CGC	CV138
sgo-1(tm2344)IV	Fig2	Colaiacovo lab	
sgo-1(sas02)IV	Fig2	This work	CEC214
osm-6(p811)V	Fig2	CGC	PR811
syIs600 [Pcol-183::mCherry; Podr-1::GFP]	Fig2	CGC	PS8438
sgo-1(tm2443)IV; syIs600 [Pcol-183::mCherry; Podr-1::GFP]	Fig2	This work	CEC278
sgo-1(tm2443)IV; sasEx06 [Psgo-1::sgo-1::sgo-1 3'UTR; Podr-1::DsRed]	Fig2	This work	CEC48
sgo-1(tm2443)IV; sasEx52 [Posm-5::sgo-1::GFP; Podr-1::DsRed]	Fig2	This work	CEC239
sgo-1(tm2443)IV; sasEx91 [Psgo-1::HA::sgo-1; ccGFP]	Fig2	This work	CEC302
unc-119(ed3)III;him-5(e1467)V;lin-2(e1309)X; sasEx49 [Posm-5::sgo-1::tdTomato; Posm-5:: tac-1::GFP; unc-119(+)]	Fig3	This work	CEC235
tac-1(or402)II	Fig4	CGC	EU1004
tac-1(or406)II ; sgo-1(tm2443)IV	Fig4	This work	CEC240
tac-1(or406)II ; sgo-1(sas02)IV	Fig4	This work	CEC241
sasEx46 [Ptac-1::tac-1::GFP::tac-1 3'UTR; rol-6 (su1006)]	Fig4, SupFig9	This work	CEC226
sgo-1(tm2344)IV;sasEx66 [Posm-5::tac-1hairpin; ccGFP] line 1	Fig4	This work	CEC259
sgo-1(tm2344)IV;sasEx67 [Posm-5::tac-1hairpin; ccGFP] line 2	Fig4	This work	CEC260
sgo-1(tm2443)IV; sasIs05 [Posm-5::tac-1::GFP::tac-1 3'UTR; Parl-13::mks-5::mCherry; rol-6(su1006)]	Fig5, SupFig11	This work	CEC246
tac-1(or406)II; sasEx74 [Posm-5::sgo-1::GFP; Parl-13::mks-5::mCherry; rol-6(su10006)]	Fig5	This work	CEC272
nxEx250 [rpi-2::GFP; mksr-1::tdTomato; rol-6(su1006)]	Fig5	Leroux lab	MX1932
sgo-1(tm2344)IV; nxEx250 [rpi-2::GFP; mksr-1::tdTomato; rol-6(su1006)]	Fig5	This work	CEC275
sasEx75 [Posm-5::sSgol1::GFP; mks-5::GFP]; ccGFP]	Fig6	This work	CEC279
sgo-1(tm2344)IV ; sasEx83 [Posm-5::sSgol1::GFP; ccGFP] line 1	Fig6	This work	CEC290
sgo-1(tm2344)IV ; sasEx84 [Posm-5::sSgol1::GFP; ccGFP] line 2	Fig6	This work	CEC291
sasEx80 [Posm-5::sgo-1::GFP; Posm-5::xbx-1::tdTomato; ccGFP]	SupFig2	This work	CEC288
gcy-9 (tm2816)	SupFig5	CGC	EAH2
sgo-1(tm2443)IV; sasEx07 [Psgo-1::sgo-1::sgo-1 3'UTR; Podr-1::DsRed] line 1	SupFig5	This work	CEC90
sgo-1(tm2443)IV; sasEx82 [Psgo-1::sgo-1::sgo-1 3'UTR; Podr-1::DsRed] line 2	SupFig5	This work	CEC91
sasEx71[Posm-5::sgo-1t^{m2443}*::mCherry; mks-3::GFP:: mks-3 3'UTR] OE line1*	SupFig7	This work	CEC264
sasEx81[Posm-5::sgo-1t^{m2443}*::mCherry; mks-3::GFP; mks-3 3'UTR] OE line 2*	SupFig7	This work	CEC266
sasEx72 [Posm-5::sgo-1::GFP; Posm-5::sgo-1^{tm2443}*::mCherry; ccGFP]*	SupFig8	This work	CEC267
unc-119(ed3)III;him-5(e1467)V;lin-2(e1309)X; sasEx57 [Posm-5::tac-1::GFP::tac-1 3'UTR; Parl-13::mks-5::mCherry; unc-119(+)]	SupFig9	This work	CEC248
unc-119(ed3)III;him-5(e1467)V;lin-2(e1309)X; sasEx58 [Posm-5::tac-1::GFP::tac-1 3'UTR; Parl-13::dyf-19::mCherry; unc-119(+)]	SupFig9	This work	CEC249
sasEx62 [Posm-5::tac-1::GFP::tac-1 3'UTR; Posm-5::tbb-4::mCherry; ccGFP]	SupFig9	This work	CEC255
nxIs30 [Psrh-220::IFT-20::gfp; cc::gfp]	SupFig10	This work	MX2426
sgo-1(tm2443)IV;nxIs30 [Psrh220::IFT-20::GFP + cc::GFP]	SupFig10	This work	MX3032
sgo-1(sas02)IV;nxIs30[Psrh220::IFT-20::GFP + cc::GFP]	SupFig10	This work	MX3039
tac-1(or402)II;nxIs30 [Psrh220::IFT-20::GFP + cc::GFP]	SupFig10	This work	MX3031
tac-1(or402)II;sgo-1(sas02)IV;nxIs30 [Psrh220::IFT-20::GFP + cc::GFP]	SupFig10	This work	MX3047
unc-119(ed3)III;him-5(e1467)V;lin-2(e1309)X; sasEx47 [Ptac-1::tac-1::GFP; Posm-5::xbx-1::tdTomato; unc-119(+)]	SupFig10	This work	CEC227
sasIs05 [Posm-5::tac-1::GFP::tac-1 3'UTR; Parl-13::mks-5::mCherry; rol-6(su1006)]	SupFig11	This work	CEC230
sasEx45 [Posm-5::tac-1::GFP::tac-1 3'UTR; Podr-1::DsRed] OE line1	SupFig12	This work	CEC244
sasEx44[Posm-5::tac-1::GFP::tac-1 3'UTR; Podr-1::DsRed] OE line 2	SupFig12	This work	CEC243
sgo-1(sas02)IV; sasEx89 [Posm-5::tac-1::GFP::tac-1 3'UTR; Parl-13::mks-5::mCherry; ccGFP] line 1	SupFig12	This work	CEC299
sgo-1(sas02)IV; sasEx90 [Posm-5::tac-1::GFP::tac-1 3'UTR; Parl-13::mks-5::mCherry; ccGFP] line 2	SupFig12	This work	CEC300
sgo-1(blt2)IV	SupFig13	Bhalla lab	-

*CGC – *Caenorhabditis* Genetics Center

Sup Table 2 – Plasmids used in this study

**Plasmid ID**	**insert**	**source**
**pCEC02**	*sgo-1 rescue (1.6kb promoter::genomic sgo-1::380bp 3'UTR)*	This work
**pCEC31**	*sgo-1 rescue (900bp promoter::HA::genomic sgo-1::unc-54 3'UTR*	
**pCEC07**	*Posm-5::sgo-1::*tdTomato*::unc-54 3'UTR*	This work
**pCEC34**	*pEntr-221-sgo-1*	This work
**pCEC35**	*pExp-32-sgo-1*	This work
**pCEC36**	*pExp-22-sgo-1*	This work
**pCEC38**	*pExp-32-tac-1*	This work
**pCEC39**	*pExp-22-tac-1*	This work
**pCEC40**	*Ptac-1::tac-1::GFP::tac-1 3'UTR*	This work
**pCEC41**	*Posm-5::tac-1::FLAG::GFP::tac-1 3'UTR*	This work
**pCEC45**	*pExp-32 N-sgo-1*	This work
**pCEC52**	*Posm-5::tbb-4::mCherry*	This work
**pCEC53**	*Posm-5::sgo-1::HA::GFP::unc-54 3'UTR*	This work
**pCEC54**	*Posm-5::tac-1hairpin::unc-54 3'UTR*	This work
**pCEC56**	*Posm-5::sgo-1(tm2443)genomic::HA::mCherry::unc-54 3'UTR*	This work
**pCEC62**	*Posm-5::sSgol1::GFP::unc-54 3'UTR5*	This work
**p328.1**	*Posm-5::xbx-1::tdTomato*	Yoder lab
**p330.1**	*mks-3::GFP*	Yoder lab
mks-5::mCherry	*Parl-13::mks-5::mCherry*	Hu lab
dyf-19::mCherry	*Parl-13::dyf-19::mCherry*	Hu lab

Sup Table 3 – Primers used in this study

**Primer ID**	**Sequence 5'-3'**	**Template**
prCC1 (F)	TCGGAAGATGCTCCAAGCCGTGCCGAC	*Psgo-1*
prCC2 (R)	GGACAACTCCAGTGAAAAGTTCTTCTCCTTTACTCATTTATAATAAATCTTCACCAATCGATACAGT	*Pgo-1/GFP*
prCC3 (F)	CTGTATCGATTGGTGAAGATTTATTATAAATGAGTAAAGGAGAAGAACTTTTCACTGGAGTTGTCC	*GFP/Psgo-1*
prCC4 (R)	ACGCGCCCTGACGGGCTTGTCTGCTCC	*GFP*
CC116F (F)	TAGCTCTCCTGTTCGCTCCT	*sgo-1* promoter
CC116R (R)	AGCAGGAAGCCGTACACATT	*sgo-1* 3' UTR
prCC83 (F)	TTGGAGGAATTGTAGCAGCA	*sgo-1* (exon1)
prCC84 (R)	TTCATTTTCGCAGATCGTTG	*sgo-1* (exon7)
prCC330 (F)	AGTCTCGTGGATAGGAAGATACT	*sgo-1* 5' UTR
prCC331 (R)	ATCTCATGCCGCTTCTTTCG	*sgo-1* 3' UTR
prCC332	CAACACATTGATGAGCAATG	*Entry clone (pENTR221-sgo-1) 5'*
prCC333	TATAACGCGTTTGGAATCACT	*5' pDEST-22*
prCC335	AGCCGACAACCTTGATTGGAGAC	*3' pDEST-22*
M-13	CAGGAAACAGCTATGAC	*Entry clone (pENTR221-sgo-1) 3'*
NestedY2Hf	GGGGACAAGTTTGTACAAAAAAGCAGGCTGGGAGGTCACCACCATGGATGCAAAAACTGCA	*Entry clone (pENTR221-sgo-1) 5'*

NestedY2Hr	GGGGACCACTTTGTACAAGAAAGCTGGGTGTCAGAAAAATGTATTGATGTATG	*Entry clone (pENTR221-sgo-1) 3'*
B1	GGGGACAAGTTTGTACAAAAAAGCAGGC**T**GGGAGGTCACCACCATGGATGCAAAAACTGCA	*Sgo-1 cDNA 5'*
B2	GGGGACCACTTTGTACAAGAAAGCTGGGTGTCAGAAAAATGTATTGATGTATG	*Sgo-1 cDNA 3'*

(F) forward; (R) reverse
